# Occurrence and Haematology of Canine Tick‐Borne Protozoa in Dhaka City, Bangladesh

**DOI:** 10.1002/vms3.70797

**Published:** 2026-01-14

**Authors:** Most. Aklima Khatun, S. M. Abdullah, Md. Kamrul Hassan, Mahfuzul Islam

**Affiliations:** ^1^ Department of Microbiology and Parasitology Faculty of Animal Science and Veterinary Medicine Sher‐e‐Bangla Agricultural University Dhaka Bangladesh

**Keywords:** blood protozoa, haematological parameters, occurrence, stray dogs, tick

## Abstract

Dogs, being companion animals, serve a variety of economic, social and cultural purposes. However, diseases caused by the tick‐borne protozoans are drawing attention globally due to their zoonotic transmission. In this context, the present study aimed to observe the occurrence of tick‐borne protozoan infections as well as some selective haematological parameters of stray dogs in Dhaka city, Bangladesh. A total of 160 dogs from various places were selected randomly and examined for both tick and protozoan infection. Only one species of hard tick, *Rhipicephalus sanguineus*, was identified, where 49 (30.62%) among the study population were found to be infested with this tick. Ticks ranging from 1–16 were removed from dogs, where most of the ticks were collected from the neck and chest regions (P < 0.001). On the other hand, examinations of blood smears confirmed three protozoan species (*Babesia canis*, *Babesia gibsoni* and *Hepatozoon* spp.) comprising 23.13% of the overall infection. Among the protozoan species, *B*. *canis* (11.88%) was the most prevalent protozoan. Subsequently, only 10.81% of the infected samples showed multiple infections. In both cases, females were more infected than males. Among the haematological parameters, the RBC counts, haemoglobin and PCV of all infected dogs were significantly lower (P < 0.001) compared to the non‐infected group. Moreover, the eosinophils of the infected groups showed higher values (11.00 % and 12.70% for protozoa and ticks, respectively) than the normal range, indicating parasitic infections. Therefore, these results suggest the necessity of frequent blood examinations to enhance animals’ welfare and disease prevention.

## Graphical abstract

• 30.62% of dogs were infested with *Rhipicephalus sanguineus*, which was mostly found on their neck and chest; however, infection with protozoa was 23.13%, where *Babesia canis* was the most prevalent (11.88%).

• RBC counts, haemoglobin and PCV were significantly lower in infected dogs with higher eosinophilic counts.

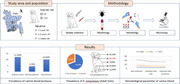

## 1 Introduction

One of the most popular pets in the world, dogs serve a variety of economic, social and cultural purposes in society (Swai et al. 2010). Keeping pet animals enhances people's self‐esteem, particularly young people (Robertson et al. 2000; Knoble et al. 2008). However, approximately 75% of dogs across the world are classified as free‐roaming (stray dogs), which has significant implications for public health (Hughes and Macdonald, 2013). Nowadays, many stray, lost, or owner‐surrendered dogs are kept in shelters to provide a temporary home until they can be reclaimed by the owner (Barrera et al. 2010). These stray animals are not even tested for parasites, vaccinated, or treated for diseases. Therefore, they serve as the reservoirs for several significant zoonotic parasites (Dakkak 2010).

These stray dogs are susceptible to different microorganisms, such as bacteria, viruses and protozoa, which can be transmitted by ectoparasites. Among them, tick‐borne protozoa are drawing attention globally for both humans and animals (Fuente et al. 2017). The brown dog tick*, R. sanguineus*, is distributed globally and is the most common in tropical areas, although it is difficult to differentiate this tick from other species having similar morphological characteristics (Walker et al. 2003) and different behaviour and ecology (Walker et al. 2000). Moreover, this tick can cause skin lesions, anaemia and tick paralysis in case of heavy infestations in dogs (Otranto et al. 2012). Furthermore, two protozoal diseases of dogs, namely babesiosis and ehrlichiosis, are transmitted by this tick (Dantas‐Torres et al. 2012). Transmission of these protozoa around the world could be due to the wide distribution of ticks (Jefferies et al. 2007; Yeagley et al. 2009).

In addition to this, the health of dogs is negatively affected by those protozoan diseases, which can result in anaemia and, sometimes, thrombocytopenia and leukopenia (Eiras et al. 2013; Piratae et al. 2017; Rautenbach et al. 2017; Thongsahuan et al. 2020). Despite this, there are very limited comprehensive studies of canine blood protozoa in the study area. Therefore, the objectives of this study were to identify different blood protozoa along with their occurrence, as well as to determine the morphological characteristics of isolated ticks from the dogs in the study areas. The study also aimed to compare the haematological profiles between infected and non‐infected dogs.

## 2 Materials and Methods

### 2.1 Ethical Approval

Blood samples were aseptically collected by registered veterinarians through proper restraining of the dogs to avoid any injuries. All the procedures required for the sample collection were fulfilled, based on the ethical guidelines approved by the Animal Welfare Act, 2019. Moreover, permission for sampling was verbally obtained from the Department of Livestock Services.

### 2.2 Study Area

**FIGURE 1:**
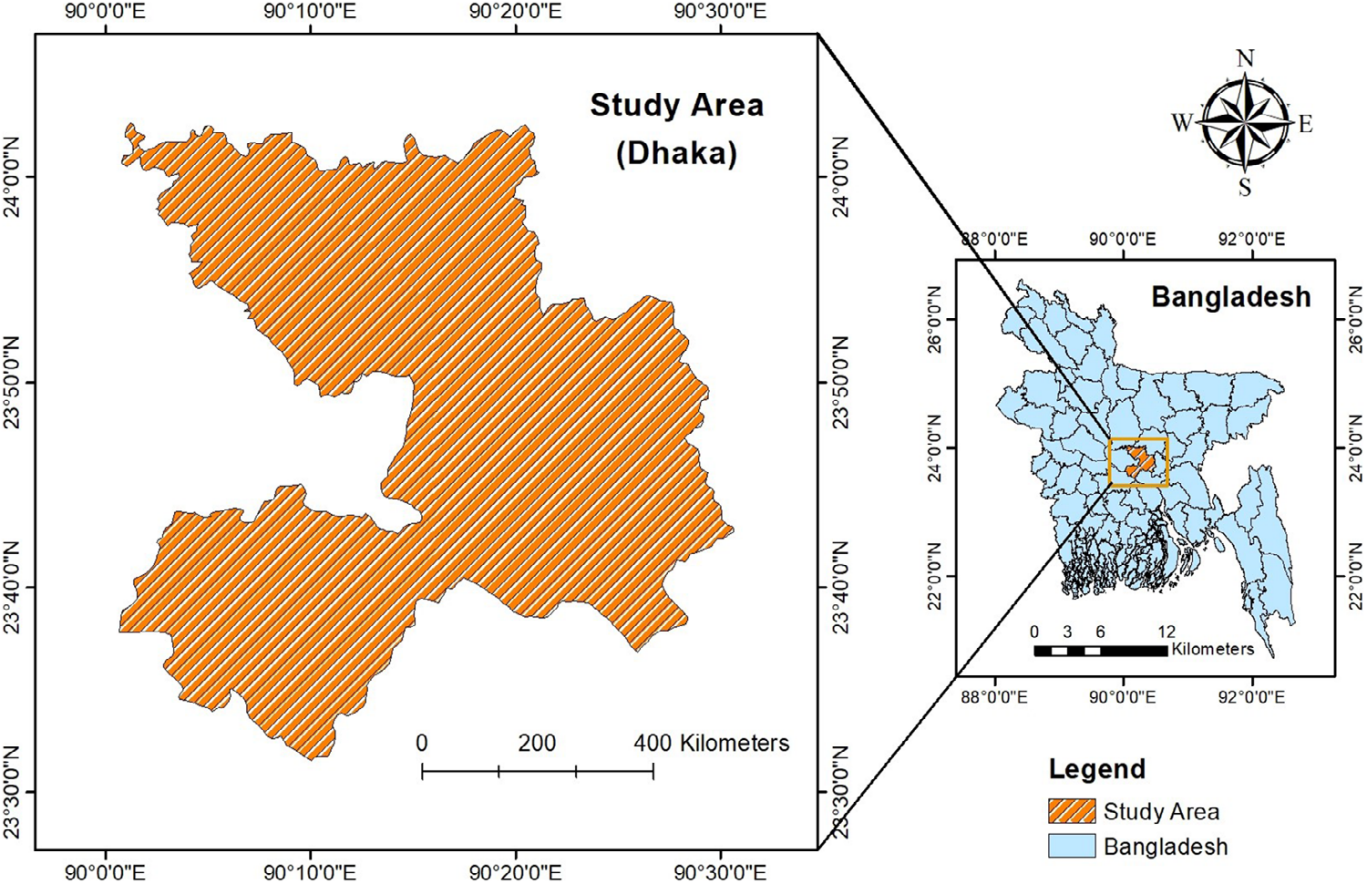
Location of study area.

This research was carried out in Dhaka, the largest city and the capital of Bangladesh (Figure 1). The city has a total area of 118.29 square miles and is situated at 23°42′N 90°22′E. Tropical vegetation covers the region, which has moist soils that are nearly flat and very near sea level. As a result of the excessive rainfall, Dhaka is vulnerable to floods during the monsoon season. The city experiences 2123 millimetres (83.6 inches) of annual rainfall and an average yearly temperature of 26°C (79°F).

### 2.3 Study Period

The cross‐sectional study lasted for six months, comprising November 2022 to April 2023, where 160 street dogs from various places were randomly selected and examined. A structured questionnaire was developed, including the tentative age, body weight, gender and so on, to collect data from the study population. During sampling, 47 individuals were male and the remaining 113 were female. Moreover, 24 dogs were of < 1 year, 52 were between 1 and 2 years and the remaining 84 were above two years of age.

### 2.4 Sample Collection

A common versatile tool, ‘Catchpole’, was used to capture and restrain the street dogs with the help of a group of trained people from the Dhaka North City Corporation (DNCC). A general anaesthesia was performed by Atropine Sulphate (0.2 mg/kg, SC) and Ketamine (2.0 mg/kg, IV) to collect both ticks and blood from the selected dogs. A comprehensive physical examination was carried out to collect ticks using forceps and placed into separate vials containing 70% alcohol with proper labelling for each dog. On the other hand, the superficial and accessible cephalic vein was used as the site for blood sampling, where 2 mL of blood was taken from each animal using a sterile 21G needle (JMI, Bangladesh) and immediately transferred to an EDTA vial (JMI, Bangladesh). Then, both vials containing ticks and blood were transferred to the laboratory, maintaining a cool chain and kept in a refrigerator (4–8°C) for further examination.

### 2.5 Blood Smear Preparation

At least 2 (two) thin smears per animal were prepared, where the cells were in a monolayer (i.e., not touching one another). A freshly prepared Giemsa working solution was made from a well‐prepared commercial stock (SRL, India) to stain blood protozoa. To conduct proper staining, a Coplin jar was filled with approximately 40 mL of Giemsa working solution (10%) and 2 drops of Triton X‐100 (Sigma‐Aldrich, USA) were added to the solution. Then, the slides were placed into the working Giemsa solution for 30 min. The excess stain was afterwards removed from those slides by dipping them three to four times in Giemsa buffer solution. Finally, the stained slides were dried by keeping them on tissue paper.

### 2.6 Tick Identification

Firstly, the ectoparasites were cleared by dissolving in 10% KOH (Merck, India) at room temperature overnight, which allowed them to pass light through them. After clearing, the specimens were returned to 50% ethanol, followed by distilled water for 30 min in each to prepare them for staining. Hematoxylin and Eosin (H & E) dye was used to stain the specimens, where the slides were kept in the stain overnight. As the specimens became darker, the excessive stain was removed by keeping them in 3% Acid‐Alcohol. Subsequently, the dehydration process was accomplished to prevent the specimen from spoiling by bacteria. Then, the specimens were cleared by xylene (Sigma‐Aldrich, USA) for a few seconds to remove ethanol and mounted in a fresh slide with Canada balsam (SRL, India). Finally, the ectoparasites (ticks) were examined under a microscope (4X and/or 10X) for morphological identification (Krantz and Walter, 2009).

### 2.7 Haematological Analysis

For haematological comparison, dogs were categorised into three groups (non‐infected, infected with ticks, and infected with protozoa), where non‐infected animals were defined as those showing the absence of both tick infestation and protozoan infection upon examination. Additionally, to ensure the uniformity among those three groups, randomly 10 blood samples from each group were analysed for different haematological parameters, such as, red blood cells (RBC), Haemoglobin (Hb), packed cell volume (PCV), mean corpuscular volume (MCV), red cell distribution width (RDW), mean corpuscular haemoglobin (MCH), MCH concentration (MCHC), white blood cells (WBC) and so on. Among these parameters, RBC and WBC were calculated in a haemocytometer slide, while Hb was detected by Sahli's method. Moreover, PCV was examined in a Wintrobe tube and recorded using the following formula.

$$\begin{array}{ccc} \mathrm{PCV}(\%) & = & (\mathrm{Height}\mathrm{of}\mathrm{packed}\mathrm{red}\mathrm{cells}\\ & & ÷\mathrm{Height}\mathrm{of}\mathrm{the}\mathrm{total}\mathrm{blood}\mathrm{in}\mathrm{the}\mathrm{tube})\times 100 \end{array}$$

Another three auxiliary indicators (MCV, MCH and MCHC), particularly in the differential diagnosis of anaemia, were recorded using the following formula (Ware 2020).

$$\begin{array}{c} \begin{array}{c} \mathrm{MCV}\left(\mathrm{fl}\right)=\left(\mathrm{PCV}÷\mathrm{Red}\mathrm{blood}\mathrm{cell}\right)\times 100\\ \mathrm{MCH}\left(\mathrm{pg}\right)=\left(\mathrm{Haemoglobin}÷\mathrm{Red}\mathrm{blood}\mathrm{cell}\right)\times 10\\ \mathrm{MCHC}\left(\%\right)=\left(\mathrm{Haemoglobin}÷\mathrm{PCV}\right)\times 100 \end{array} \end{array}$$

### 2.8 Statistical Methods

The obtained data were imported, stored and coded accordingly using Microsoft Excel 2016, where all data analyses were performed by using a statistical software program (SPSS for Windows, Version 19.0, USA). The results of occurrence were expressed as a percentage. Association among various risk factors, namely, sex, age and season, was carried out by Chi‐square (𝜒2‐test). Moreover, the standard error of the mean was also determined for haematological parameters.

## 3 Results

### 3.1 Morphological Identifications of Blood Protozoa

**FIGURE 2:**
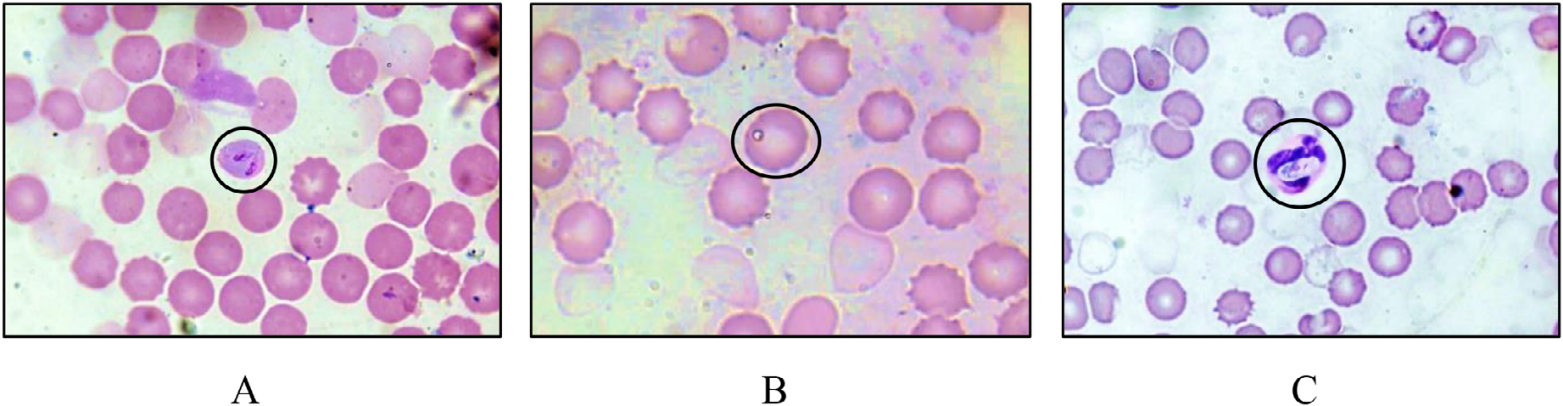
Microscopic observation of canine blood protozoa (100X magnifications). (A) *Babesia canis*; (B) *Babesia gibsoni*; and (C) *Hepatozoon* spp. (black circle indicates the organism).

*Babesia* species can be identified by their orientation in RBCs, where *B. canis* makes an acute angle, while *B. gibsoni* appears single in most cases. In our study, the shape of *B. canis* was observed as pyriform, where one end was pointed and the other end was rounded (Figure 2A). On the other hand, *B. gibsoni* lacked the usual pyriform shapes and had a signet ring form (Figure 2B). Moreover, in the stained blood smear under the microscope, *Hepatozoon* spp. was easily identified in the cytoplasm of WBCs (mostly in neutrophils) where the gamonts were observed elongated, ellipsoidal and had an eccentrically positioned nucleus (Figure 2C).

### 3.2 Morphological Identifications of Rhipicephalus sanguineus

**FIGURE 3:**
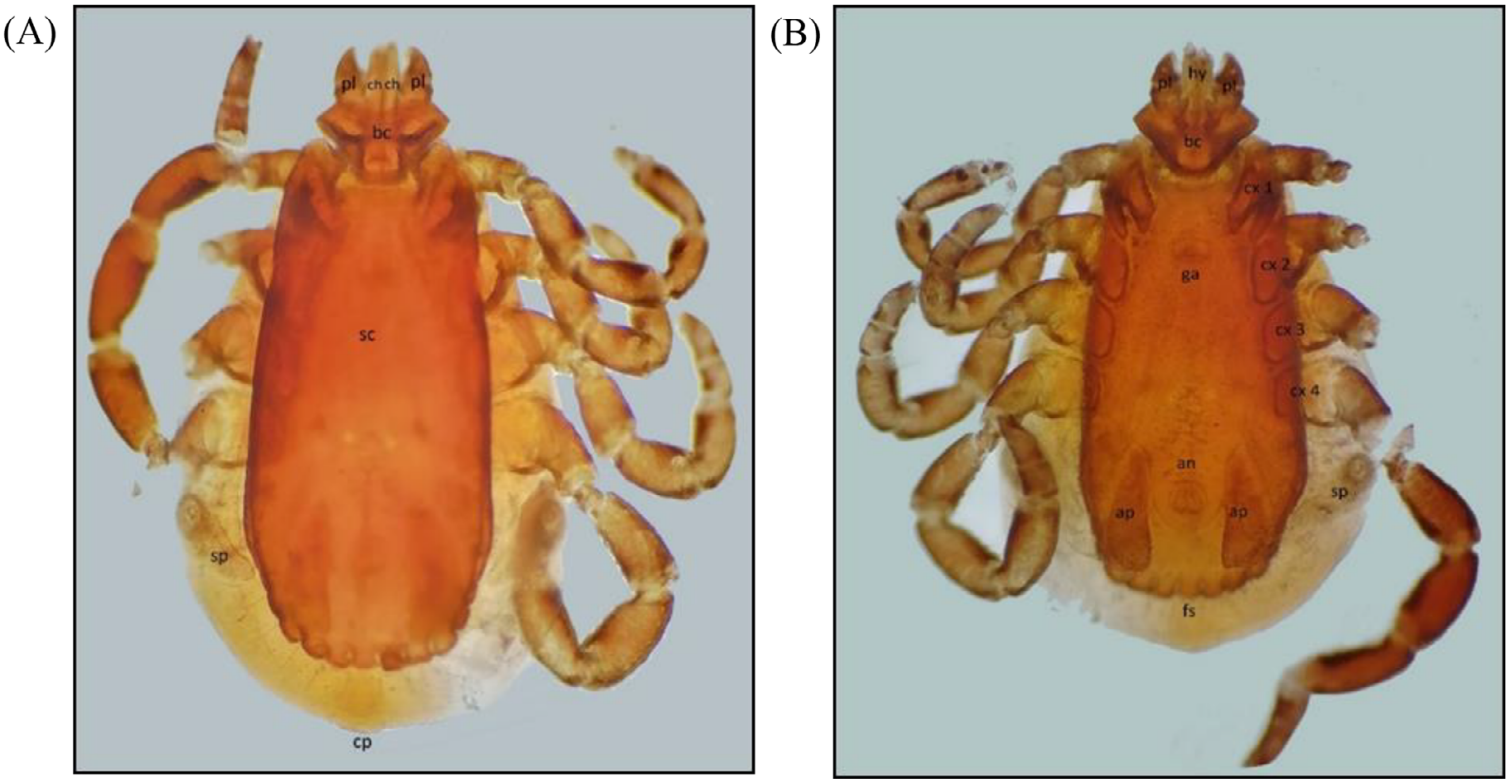
Male *Rhipicephalus sanguineus* (4X); dorsal view (A) & ventral view (B). pl, palp; hy, hypostome; ch, chelicerae; bc, basis capitulam; cx, coxa; sc, scutum; sp, spiracle; an, anus; ap, adanal plate; fs, festoon; ga, genital apron; cp, caudal plate.

Medium‐sized, yellowish‐brown to reddish‐brown ticks having a dark, inornate brown scutum were measured with a scale, where unfed males and females were found on an average of 3.60 and 4.23 mm, respectively. The capitulum, or anterior portion of the body (Figure 3), was composed of one hexagonal‐shaped basis capitulum, which supports several organs, such as one powerful hypostome for sucking blood, two chelicerae for cutting the skin and two short palps for sensory function. Furthermore, in all the studied specimens in our investigation, setae and sensilla were discovered to be present throughout the body without a distinct pattern of distribution.

Despite intraspecific heterogeneity among the study samples, festoons were located on the posterior margin of the body and were separated into 11 unique rectangular portions (Figure 3). The size of the caudal process varied across the specimens under study in fed males. The two valves and an anal groove that created the anal orifice were articulated with four setae that are positioned symmetrically on each side. Adanal plates and accessory shields were found in male ticks, composing the anogenital region. These structures are highly sclerotised and located on the side of the anus. The adanal plates were long, parallel and had a sharp posterior margin. The accessory shields, varying in form, were located beside the adanal plates. The narrow spiracular plates were located behind the last pair of legs. A circular ostial lip was also present in each spiracular plate. The genital plate was located between the 1^st^ and 2^nd^ pair of coxae and exhibited a round structure in all the specimens in our study.

### 3.3 Occurrence of Blood Protozoa

#### 3.3.1 Overall Occurrence of Blood Protozoa

**FIGURE 4:**
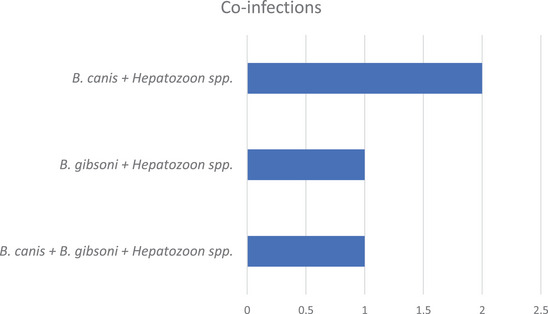
The occurrence of co‐infections with canine blood protozoa.

Examination of blood smear was performed under a light microscope, where 37 out of 160 samples (23.13%) were infected with any of the three species of blood protozoa, namely *Babesia canis*, *Babesia gibsoni* and *Hepatozoon* spp. In addition to this, the species‐wise occurrence of canine blood protozoa showed statistically significant differences, where *B. canis*, *B. gibsoni* and *Hepatozoon* spp. were detected in 19, 7 and 16 dogs, comprising 11.88%, 4.38% and 10.00%, respectively (Table 1). However, of these 37 positive dogs, 33 (89.19%) were infected with only one species, while infections with more than one protozoan were found only in 4 (10.81%) dogs (Table 2). These mixed infections were observed with two (2) and three (3) species of canine blood protozoa, comprising 8.11% and 2.70%, respectively. These single and mixed infections of protozoa had a statistical significance (P <0.001). Moreover, the occurrence of these co‐infections with canine blood protozoa is exhibited in Figure 4.

**TABLE 1:** Species‐wise occurrence of canine blood protozoa.

Species	No. of dogs infected (n = 160)	Occurrence %	*p*‐value
*Babesia canis*	19	11.88	0.047^*^
*Babesia gibsoni*	7	4.38	
*Hepatozoon* spp.	16	10.00	

* indicates statistically significant.

**TABLE 2:** Occurrence of single and mixed infections of protozoa.

Types of infection	No. of dogs infected (n = 37)	Occurrence %	*p*‐value
Single infection	33	89.19	< 0.001^*^
Multiple infections			
Two species	3	8.11	
More than two species	1	2.70	

^*^ Indicates statistically significant.

#### 3.3.2 Occurrence of Blood Protozoa in Relation to Different Risk Factors

The proportion of gender was mentioned before, where 70.63% were females and the rest 29.37% were males. In the case of gender, a little difference was observed where females (24.78%) were infected with canine blood protozoa more than males (19.15%). However, all dogs were categorised into three groups (i.e., < 1 year, 1–2 years and > 2 years), where the highest occurrence (29.17%) was seen in the younger groups of age (< 1 year), followed by 23.08% in the age group of 1–2 years and 21.43% in the age group of more than 2 years. These results indicated more canine blood protozoan infections in puppies than in adults. Protozoan infection in relation to gender and age is shown in Table 3.

**TABLE 3:** Occurrence of canine blood protozoa in relation to different risk factors.

Group	Variable	No. of examined	No. of infected	Occurrence %	*p*‐value
Gender	Male	47	9	19.15	0.442
	Female	113	28	24.78	
Age	< 1 year of age	24	7	29.17	0.730
	1–2 years of age	52	12	23.08	
	> 2 years of age	84	18	21.43	

#### 3.3.3 Occurrence of Canine Blood Protozoa in Different Locations

**FIGURE 5:**
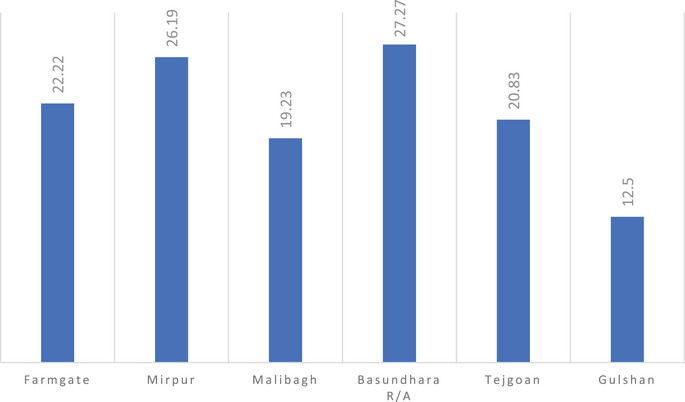
Occurrence of canine blood protozoa in different locations.

As mentioned before, a total of 160 stray dogs from 6 different locations in Dhaka city were included in this study. Microscopic examination of the blood revealed the highest incidence in Basundhara R/A (27.27%), followed by Mirpur (26.19%), Farmgate (22.22%), Tejgaon (20.83%), Malibagh (19.23%) and Gulshan (12.50%). This area‐wise infection of blood protozoa is given in Figure 5.

### 3.4 Occurrence of Ticks

#### 3.4.1 Overall Occurrence of Adult Ticks in Dhaka City

A total of 160 stray dogs, varying in age and sex, were selected and examined for ticks after performing general anaesthesia. Upon visual inspection, 49 of those study populations were found to be infested with brown dog tick, *Rhipicephalus sanguineus*. Area‐wise overall occurrence (30.62%) is shown in Table 4. Concerning area‐wise infestation, the highest infection was encountered in Basundhara R/A (42.42%), followed by Malibagh (30.77%), Farmgate (29.63%), Mirpur (28.57%), Tejgoan (25.00%) and Gulshan (12.50%). The infected dogs produced a total of 278 adult ticks, where the average sex ratio was 3.21, indicating more female ticks on the host.

**TABLE 4:** Overall occurrence of adult ticks in Dhaka city.

Areas	No. of examined	No. of infected	Occurrence%	Collected female ticks	Collected male ticks	Sex ratio
Farmgate	27	8	29.63	38	12	3.16
Mirpur	42	12	28.57	52	19	2.73
Malibagh	26	8	30.77	28	7	4.00
Basundhara R/A	33	14	42.42	62	18	3.44
Tejgoan	24	6	25.00	29	8	3.62
Gulshan	8	1	12.50	3	2	1.50
**Total**	**160**	**49**	**30.62**	**212**	**66**	**3.21**

#### 3.4.2 Occurrence of Ticks in Relation to Different Risk Factors

Among the examined 47 male dogs, 10 (21.28%) were found infested with ticks. On the other hand, 39 dogs out of 113 female dogs, comprising 34.51% of the total population, were found to be infested with ticks during the study. Considerably, females were infected more than the male dogs, where there was no statistical difference. Moreover, the occurrence of tick infestation varied depending on the age of the studied samples. Dogs aged less than 1 year showed the highest infection (37.50%) followed by 1–2 years (28.85%) and more than 2 years (29.76%) age groups. From these results, it was clear that young dogs were affected by ticks more than adults, although there was no statistical significance among them. These variations in relation to different risk factors are given in Table 5.

**TABLE 5:** Occurrence of ticks in relation to different risk factors.

Group	Variable	No. of examined	No. of infected	Occurrence %	*p*‐value
Gender	Male	47	10	21.28	0.098
	Female	113	39	34.51	
Age	< 1 year of age	24	9	37.50	0.726
	1–2 years of age	52	15	28.85	
	> 2 years of age	84	25	29.76	

#### 3.4.3 Seasonal Occurrence of Ticks

The present study was initiated in November 2022, when we collected samples from dogs. Since then, the collection of ticks from dogs has continued till April 2023. Therefore, only two major seasons, namely winter (November–February) and summer (March–April), were included in our study. There were differences in tick burdens in different seasons, with higher infestation levels in summer (37.50%) followed by winter (26.92%). Moreover, the monthly incidence of tick infestation is given in Table 6, where the highest percentage of tick‐infested dogs were examined in March (37.84%) and the lowest percentage was marked in December (23.81%).

**TABLE 6:** Seasonal distribution of ticks in infected dogs.

Months	No. of examined	No. of infected	Occurrence %	*p*‐value	Seasons	Occurrence %	*p*‐value
November	32	9	28.13	0.787	Winter	26.92	0.166
December	21	5	23.81				
January	31	8	25.81				
February	20	6	30.00				
March	37	13	35.84		Summer	37.50	
April	19	8	42.11				

#### 3.4.4 Degree of Tick Infestation

In our study, ticks were removed individually from each dog, with an average of 1–16 ticks per dog. To determine the degree of tick infestation, a total of 4 categories were identified: first, low infestation rate comprising 1–4 numbers of ticks, second, mild infestation rate comprising 5–8 numbers of ticks, third, moderate infestation rate comprising 9–12 numbers of ticks and finally, high infestation rate comprising 13–16 numbers of ticks. The degree of tick infestation having a statistical significance is included in Table 7.

**TABLE 7:** Determination of the degree of tick infestation.

Degree of infestation	No. of ticks counted	No. of dogs infected	Occurrence %	*p*‐value
Low (+)	1–4	22	44.89	0.001^*^
Mild (++)	5–8	16	32.65	
Moderate (+++)	9–12	7	14.28	
High (++++)	13–16	4	8.16	

^*^ Indicates statistically significant.

#### 3.4.5 Regions of Infestation by Ticks on Dogs’ Bodies

Predominantly, five (5) regions were identified on the dog's body after reviewing the literature and these areas were the head with ears, neck and chest regions, back region, abdomen and legs. As we mentioned before, 278 adult ticks were collected during this study, while 95 ticks, comprising nearly one‐third of the total population, were found around the neck and chest region, which expressed the highest percentage of other parts of the body with a statistical significance (P < 0.001). On the other hand, the back region comprised the lowest percentage of the availability of ticks. The attachment of ticks on the host's body is exhibited in Table 8.

**TABLE 8:** Attachment of ticks on the host's body.

Attachment of ticks	No. of ticks counted	Occurrence %	*p*‐value
Head with ears	56	20.14	< 0.001^*^
Neck and chest region	95	34.17	
Back region	19	6.83	
Abdomen	72	25.89	
Legs	36	12.94	

^*^ Indicates statistically significant.

### 3.5 Haematological Parameters of Blood

Of the 160 samples collected from dogs, 49 were positive for *R. sanguineus* and 37 were positive for different protozoan infections. The average haematological values obtained from the non‐infected and infected groups are presented in Table 9. The RBC counts, Hb and PCV of all infected dogs were numerically lower compared to the non‐infected group. The average values RBC (6.23, 4.51 and 4.69), Hb (15.48, 11.44 and 11.18) and PCV (43.80, 33.50 and 32.10) were recorded from the non‐infected group, the infected group with protozoa and the infected group with ticks, respectively, which indicated the different degrees of anaemia. The average values of MCV, MCH and MCHC were found in the normal range. On the other hand, the average WBC count was higher in the infected groups, where the average value of WBC for protozoan was 16.90 × 10^3^ cells/µL and the average value of WBC for tick infestation was 17.30 × 10^3^ cells/µL. When compared to the different leukocyte counts, the eosinophils of the infected groups showed higher values (11.00 % and 12.70% for protozoa and ticks, respectively) than the normal range, indicating the parasitic infections.

**TABLE 9:** Hematological profiles of dogs infected with protozoa and ticks compared to healthy dogs.

Parameters	Non‐infected animals	Infected with protozoa	Infected with ticks	SEM	*p*‐value	Reference value
RBC (10^6^ cells/µL)	6.23^a^	4.51^b^	4.69^b^	0.081	< 0.001^*^	5.5–8.5
Hb (g/dL)	15.48^a^	11.44^b^	11.18^b^	0.265	< 0.001^*^	12–19
PCV (%)	43.80^a^	33.50^b^	32.10^b^	0.901	< 0.001^*^	37–57
MCV (fL)	70.34	74.78	68.56	2.140	0.143	66–77
MCH (pg)	24.87	25.49	23.89	0.639	0.238	19.5–24.5
MCHC (%)	35.43	34.24	35.06	0.857	0.662	32–36
WBC (10^3^ cells/µL)	13.66^b^	16.90^a^	17.30^a^	0.295	< 0.001^*^	6–17
Neutrophil (%)	70.70^a^	66.40^b^	65.90^b^	1.244	0.024	58–85
Lymphocyte (%)	14.50	13.40	11.30	0.928	0.065	8–21
Monocyte (%)	8.80	9.20	10.20	0.460	0.134	2–10
Eosinophil (%)	6.00^b^	11.00^a^	12.70^a^	0.673	< 0.001^*^	0–9

*Note*: ^a or b^ in the same row indicates the significant differences (p < 0.05) of data among different groups of dogs.

* Statistically significant.

Abbreviations: Hb = hemoglobin, MCH = mean corpuscular hemoglobin, MCHC = mean corpuscular hemoglobin concentration, MCV = mean corpuscular volume, PCV = packed cell volume, RBC = red blood cells, WBC = White blood cells, SEM = Standard error of the mean.

## 4 Discussion

A total of 160 street dogs were included in this study, where samples were collected, smeared and stained for microscopic identification following a proper scientific method. Three (3) protozoan species, namely, *B. canis*, *B. gibsoni* and *Hepatozoon* spp., were identified according to the keys and descriptions of various authors (Allison and Little 2013; Saari et al. 2019). On the other hand, only one species of ticks, *R. sanguineus*, was identified according to various authors (Walker et al. 2000; Guglielmone et al. 2006; Krantz and Walter 2009; Dantas‐Torres et al. 2013; Nava et al. 2015).

Moreover, nearly one‐fourth (23.13%) of the study samples were infected with at least one protozoon, which is very similar to various reports in Southeast Asia where the prevalence of canine blood protozoan infections reached up to 28% (Laummaunwai et al. 2014; Sontigun et al. 2022). However, other authors (Piratae et al. 2017; Juasook et al. 2021) revealed higher prevalence than our study. These higher infection rates may be due to the difference in methodology, where most of them applied molecular techniques that provide more specific genetic and species information (Sainz et al. 2015; Das et al. 2020). Notably, *B. canis* was encountered in the highest number found during the study, which is similar to the studies in the above‐mentioned areas, including the Indian Sub‐continent (Singh et al. 2014; Jain et al. 2017). Although Piratae et al. 2017 and Thongsahuan et al. 2020 found more infections with *Hepatozoon* spp. than *Babesia*. The variation in species‐wise prevalence might be due to the geographic location, distribution of vectors, methods of sampling, etc. (Singla et al. 2016; Rucksaken et al. 2019). Furthermore, the results of mixed infections are supported by Mylonakis et al. (2004), Kumar and Varshney (2007) and Yabsley et al. (2008). In our study, the proportion of gender in the observed population was nearly 2:1, where the females were 70.63% and the rest 29.37% were males. In the case of gender, females were more infected than males and this result is inconsistent with that of Amuta et al. (2010) and Singh et al. (2011). The physiological stress experienced by females during nursing, oestrus and pregnancy may be the cause of this greater incidence. Moreover, the occurrence of blood protozoa was found to be highest in young dogs, due to various risk variables including immunity, habitat, interaction and so on. (Samradhni et al. 2005). Co‐infections with canine blood protozoa are thought to arise because of the research area's warm, humid urban climate, as well as the large number of stray dogs and the frequent encounters between humans and animals. It is probable that the affected dogs were mostly free‐roaming, varied in age and sex and had minimal access to veterinary treatment, which increases their susceptibility to various protozoa (Boonhoh et al. 2023).

During our study, a total of 278 adult ticks were gathered from the infected dogs, where the average sex ratio was 3.21, indicating more female ticks on the host. The sex ratio is in complete disagreement with Dantas‐Torres and Otranto 2011 who reported more male ticks. This variation may be due to the longer attachment time of females than males (Handeland et al. 2013). This study revealed a moderate infestation of ticks in dogs sampled in Dhaka city and the percentage was 30.62%, which was very similar to Zeb et al. (2023), conducted in Pakistan. However, the results of this study showed a lower infection rate than the neighbouring countries, where the prevalence of ticks in India, Pakistan and Indonesia has been reported at 45.0%, 53% and 67.9%, respectively (Bhadesiya et al. 2014; Soundararajan 2016; Grant et al. 2023). On the other hand, Shimada et al. 2003, Ul‐Hasan et al. 2012 and Saleh et al. 2019 reported a much lower prevalence than the present study. This fluctuation in prevalence might be brought on by factors such as climate, geographic distribution, sample size, methods of sample collection, etc (Apanaskevich and Oliver 2014). Considering the gender‐wise occurrence of ticks, females were heavily infected more than the male dogs due to their prolonged sitting habit on the ground and nursing their puppies (James‐Rugu and Jidayi 2004). This study showed young dogs were affected with more ticks than adults, which may be related to gradual immunity development and close proximity to the ground (Abdulkareem et al. 2018). Seasonal variances in tick infestation, higher in summer, could be attributed to the warm and humid environment accelerates their development and reproduction (Bouattour 2002). In addition to this, the neck and chest appeared to be the most favoured preference sites for ticks on dogs, which is supported by Foldvari and Farkas (2005).

Canine babesiosis and hepatozoonosis are important tick‐borne diseases that infect dogs worldwide. The results of this study indicated that the presence of both protozoa and ticks was considered as risk factors, showing significantly lower RBC, Hb and PCV volumes. The results from RBC parameters indicated normocytic normochromic anaemia, which is non‐regenerative due to bone marrow dysfunction (Fleischman 2012). These RBC indices, which were computed from blood samples infected with both protozoa and ticks, were below the accepted reference limits and consistent with previously published findings (Das and Konar 2013; Wongsawang and Jeimthaweeboon 2018; Piratae et al. 2019). In fact, anaemia is a common finding in canine blood protozoan infection, which occasionally can be severe (Das and Konar 2013; Bhadesiya and Raval 2015; Wongsawang and Jeimthaweeboon 2018). Moreover, WBC abnormalities were also found in protozoa and tick‐infected dogs compared to the non‐infected ones, which agreed with Paiz et al. (2016) and Wongsawang and Jeimthaweeboon (2018). Although molecular and serological methods have increased sensitivity and specificity, microscopy persists as one of the most widely used techniques for detecting blood protozoa due to its quick and simple technique. It allows identification of parasites within stained blood smears through direct visualisation and enables the detection of morphological features to distinguish different species (de Waal 2012).

## 5 Conclusion

Ticks are considered one of the important obligate blood‐sucking arthropods after mosquitoes. Several ecological parameters, including seasonal variations, temperature, relative humidity and vegetation, are linked to the transmission of these ectoparasites. Our study revealed three species of blood protozoa (*B. canis*, *B. gibsoni* and *Hepatozoon* spp.) and only one species of ticks (*R. sanguineus*) from the study area. Furthermore, haematological abnormalities (low RBC, Hb and PCV) were strongly associated with blood protozoan infections. These findings underscore the necessity of monitoring the haematological parameters for the clinical diagnosis of blood protozoa.

## Author Contributions

**Most. Aklima Khatun**: conceptualisation, methodology, data curation, formal analysis, funding acquisition, writing – original draft, writing – review and editing. **S. M. Abdullah**: conceptualisation, methodology, data curation, formal analysis, writing – original draft, writing – review and editing. **Md. Kamrul Hassan**: data curation, supervision, writing – review and editing. **Mahfuzul Islam**: conceptualisation, data curation, validation, funding acquisition, supervision, writing – review and editing.

## Funding

This study was funded by the National Science and Technology (NST) grant from the Ministry of Science and Technology, Government of the People's Republic of Bangladesh (Grant Number: 2022–2023/111).

## Conflicts of Interest

The authors declare no conflicts of interest.

## Data Availability

The data that support the findings of this study are available on request from the corresponding author.
